# Improvement of the Management of Infants, Children and Adults with a Molecular Diagnosis of Enterovirus Meningitis during Two Observational Study Periods

**DOI:** 10.1371/journal.pone.0068571

**Published:** 2013-07-11

**Authors:** Christine Archimbaud, Lemlih Ouchchane, Audrey Mirand, Martine Chambon, François Demeocq, André Labbé, Henri Laurichesse, Jeannot Schmidt, Pierre Clavelou, Olivier Aumaître, Christel Regagnon, Jean-Luc Bailly, Cécile Henquell, Hélène Peigue-Lafeuille

**Affiliations:** 1 Université d’Auvergne, Laboratoire de Virologie - EPIE EA4843, Clermont-Ferrand, France; 2 CHU Clermont-Ferrand, Laboratoire de Virologie - Centre National de Référence des Entérovirus/Parechovirus, Laboratoire Associé, Clermont-Ferrand, France; 3 Université d’Auvergne, Service de Biostatistiques, Clermont-Ferrand, France; 4 CHU Clermont-Ferrand, Service de Pédiatrie Générale, INSERM CIC 501, Clermont-Ferrand, France; 5 CHU Clermont-Ferrand, Service des Maladies infectieuses, Clermont-Ferrand, France; 6 CHU Clermont-Ferrand, Service Accueil Urgences, Clermont-Ferrand, France; 7 CHU Clermont-Ferrand, Service de Neurologie, Clermont-Ferrand, France; 8 CHU Clermont-Ferrand, Service de Médecine Interne, Clermont-Ferrand, France; University of North Carolina School of Medicine, United States of America

## Abstract

Enteroviruses (EVs) are a major cause of aseptic meningitis, and RNA detection using molecular assay is the gold standard diagnostic test. The aim of this study was to assess the impact of an EV positive diagnosis on the clinical management of patients admitted for meningitis over the course of two observational study periods (2005 and 2008–09) in the same clinical departments. We further investigated in multivariate analysis various factors possibly associated with hospital length of stay (LOS) in all age groups (infants, children, and adults). The results showed an overall improvement in the management of patients (n = 142) between the study periods, resulting in a significantly shorter hospital LOS for adults and children, and a shorter duration of antibiotic use for adults and infants. In multivariate analysis, we observed that the time from molecular test results to discharge of patients and the median duration of antibiotic treatment were associated with an increase in LOS in all age groups. In addition, among adults, the turnaround time of the molecular assay was significantly correlated with LOS. The use of CT scan in children and hospital admission outside the peak of EV prevalence in infants tended to increase LOS. In conclusion, the shorter length of stay of patients with meningitis in this study was due to various factors including the rapidity of the EV molecular test (particularly in adults), greater physician responsiveness after a positive result (in adults and children), and greater experience on the part of physicians in handling EV meningitis, as evidenced by the shorter duration of antibiotic use in adults and infants.

## Introduction

Enteroviruses (EVs), the most common cause of viral meningitis in the pediatric population, are also present in adults [1], [2], [3], [4]. They are responsible for annual seasonal epidemics (summer and fall) of aseptic meningitis in temperate climates, and are maintained at low but detectable levels during the cold months [3], [5]. It is now widely agreed that nucleic acid amplification tests for enterovirus (EV) RNA in cerebrospinal fluid (CSF) specimens is the “gold standard” for the diagnosis of enteroviral meningitis [3], [6], [7], [8]. These molecular assays can provide rapid results within 5–24 hr of receipt of the sample. Numerous studies, mainly performed in infants and children, have shown that the prospective use of molecular tests on admission, on the sole basis of febrile state in infants or clinical suspicion of meningitis, has improved management by reducing hospital length of stay (LOS), use of antibiotics, and hospital-related costs [3], [4], [9], [10], [11].

The assessment of professional practices is becoming increasingly a part of the accreditation procedures for healthcare establishments. In a previous study, we showed that the management of patients with EV meningitis diagnosed with positive RT-PCR test result differed between infants, children, and adults admitted to the University Hospital of Clermont-Ferrand (France) in 2005 [3]. The mean length of stay (LOS) for adults or infants was significantly longer than for children and was correlated with more frequent prescription of antibiotics and acyclovir.

The aim of this study was therefore to assess the clinical management of patients (infants, children, and adults) with proven EV meningitis between two periods fairly close together (2005 and 2008–2009) with a particular focus on viral diagnosis (test turnaround time, [TAT]), hospital LOS, time of patient discharge, use of antibiotics and acyclovir, and computer tomography (CT) scan.

## Materials and Methods

### Ethics Statement

The study was approved by the Committee for the Protection of Human Subjects (Comité d’Ethique des Centres d’Investigation Clinique de l’inter-région Rhône-Alpes-Auvergne), with a waiver of informed consent (IRB number 5044).

### Study Design

The study was conducted at the University Hospital of Clermont-Ferrand (France) in consecutive patients admitted for suspected meningitis between January 1, 2008 and December 31, 2009. We prospectively analyzed their CSF specimens using a real-time EV molecular assay irrespective of cytological examination results [12]. Patients were eligible for inclusion in the study on condition of a positive detection of EV genome in CSF by an EV molecular assay (see below). In patients with proven enteroviral meningitis, data were extracted from medical charts and hospital computer records. A standardized questionnaire was used to collect information related to patients’ clinical history, demographic information, CSF parameters, the use of cranial CT scan, and administration of antibiotic or antiviral treatments. The date and time of patients’ admission, of the arrival of CSF samples in the laboratory, of the availability of results, and hospital LOS were also collected. All the data were compared with those obtained in a previous study of 69 patients admitted between January 1 and December 31, 2005 to the same hospital [3]. The EV season was defined as May 1 through October 31 for each year.

### Patients and Samples

The University Hospital of Clermont-Ferrand is composed of two geographically separate institutions. The first includes the units of adult emergency medicine, neurology, internal medicine and infectious diseases and is close to the virology laboratory. The second includes the pediatric emergency unit and pediatric wards and is about 30 minutes’ drive from the laboratory. A single virology laboratory performs all virological analyses.Of the 887 consecutive patients admitted to the hospital for suspected meningitis, between January 1, 2008 and December 31, 2009, 73 had proven EV meningitis by positive molecular test. This population of 73 patients was stratified into age groups: infants ≤2 years (n = 17, 23.3%; median age 32 days [9–458 days]); children from 2 to 16 years (n = 23, 31.5%; median age 7 years [4–14 years]); and adults ≥16 years (n = 33, 45.2%; median age 31years [16–57 years]). Leucocytosis was defined as a CSF white blood cell (WBC) count >19 cell/mm^3^ if the patient’s age was <28 days, and ≥10 cell/mm^3^ for older patients [13]. All the patients underwent bacteriological investigations (including cytological examination, Gram stain and culture), and tested negative. Virological investigations (molecular detection of herpes simplex virus with the NucliSens EasyQ HSV1/2 assay BioMérieux, France), performed in 54/73 patients (including all the adults), were also negative.

### Real-time Amplification EV RNA Assay

During the two study periods, CSF samples were analyzed using an EV real-time assay 5 days a week from Monday to Friday throughout the year. In 2008–09, viral RNA was extracted from CSF samples (200 µl) by a NucliSens® EasyMAG™ extractor (bioMérieux, Marcy l’Etoile, France) and was recovered in 25 µl of the elution solution. Subsequently the real-time nucleic acid sequence based amplification (NASBA) EV assay was performed with NucliSens EasyQ Enterovirus reagents (bioMérieux) according to the manufacturer’s instructions. In 2005, the viral RNAs were extracted manually with the QIAamp viral RNA mini kit (Qiagen, Courtaboeuf, France), and amplification was performed with an in-house one-step real-time RT-PCR assay as described previously [6]. These two molecular amplification assays and analysis (including the extraction step) took about 3 hours. The results were given by phone to the patients’ attending physician as soon as they were available.

### Statistical Analysis

The data were organized in a database for the purposes of statistical analysis. We calculated the molecular test TAT (date and time molecular test result was reported minus date and time of the arrival of CSF sample in the laboratory), molecular test-to-discharge time (date and time of discharge minus date and time of test result), and length of stay (LOS; date and time of discharge minus date and time of admission).

Continuous variables were displayed either as mean ± SD or median and interquartile range (IQR). Categorical variables were described by using frequencies and percentages. Comparisons of patient management in the two study periods (2005 versus 2008–09) were performed with Wilcoxon rank sum tests for continuous variables. Categorical variables were described using the Pearson chi-square test, Fisher exact test (if needed), and odds ratios (OR) with their 95% confidence intervals (95% CI). Separate analyses were performed in infants, children and adults to investigate possible relationships between LOS and other variables. This was done because of the occurrence of non-specific clinical symptoms in infants and to enable comparisons with previous studies, which are mainly restricted to infants or children. Pearson’s and Spearman’s correlation coefficient tests were performed to compare hospital LOS and quantitative variables including duration of antibiotic or antiviral treatments, TAT, and time to discharge from EV molecular test results. LOS was considered as time to event, i.e. time to discharge from hospital admission. Univariate time to event analyses were performed using homogeneity tests (Log-Rank test and Wilcoxon generalized test) and computing crude and adjusted hazard ratios (HR) with 95% CI through a simple Cox regression model which required TAT and time to discharge from molecular results to be transformed into ordinal classes. TAT was separated into four ordinal categories: (1) results available on the day, (2) results available within 24 hours and (3 and 4) results available within or after 48 hours. Test-to-discharge time was separated into four ordinal categories: (1) discharge after 1 to 5 h after results, (2) discharge within 6–24 h after results, (3) discharge >24 h after results and (4) discharge before results available. An HR over 1 denoted a decrease in LOS. Multivariate analyses used a Cox regression model including a forward-stepwise selection procedure (p<0.2 to entry and p<0.05 to stay in the model) except for gender and period of study, which were kept for adjustment in the model as potential confounders. SAS v9.3 (SAS institute inc., Cary, NC, USA) was used for data analysis with a two-sided type I error set at 0.05.

## Results

### Management of Patients with Enterovirus Meningitis in 2008–09

Between January 1, 2008 and December 31, 2009, EV-proven meningitis was diagnosed in 73 patients, of whom 67 (92%) were admitted within the EV season. CSF pleocytosis was present in 58/73 patients (79%; range 10–848; median 87 WBC/mm3), mainly in children (22/23, 96%) and adults (29/33, 88%), and only in 7 (41%) infants.

Because the EV molecular test is performed 5 days a week throughout the year, only the 52 patients admitted between Sunday and Friday morning were included to investigate the impact of the test on patient management (Table 1). The median EV molecular test turnaround time (TAT) was about 1 day for infants and children and 0.4 days for adults (p = 0.33). The results of the EV molecular test were available on the same day, within the first 24 hr, and within 48 hr after the arrival of the CSF in the lab for 56%, 92%, and 98% of patients, respectively. Of the 52 patients, 10/11 (91%) infants, 20/25 (80%) adults and 7/16 (44%) children had a positive EV assay result available prior to discharge. With regard to the clinical management of patients, the median [interquartile range] test-to-discharge time was significantly longer in infants and adults (22 hr [19–42] and 19 hr [11–21], respectively) than in children (−0.3 hr [(−)5 - 1]) (p<0.05). The negative value observed in children means the patient had left the hospital before the clinician received the results. A total of 86% of children, 5% of adults, and no infants were discharged within 1 and 5 hr after the positive results were transmitted to the clinicians. Within 24 hours, 14% of children, 70% of adults, and 70% of infants were discharged.

**Table 1:** Comparison of time parameters and service use in infants, children, and adults with enterovirus meningitis in 2008–09 with those of a previous observational study in 2005^a^.

	Infants			Children			Adults		
	2005	2008–09	P value	2005	2008–09	P value	2005	2008–09	P value
	n = 8	n = 17		n = 45	n = 23		n = 16	n = 33	
**Turnaround time of molecular test (TAT)** *									
**median [IQ25–IQ75], days** b	1.9 [1.1–3.3]	1 [0.4–1.2]	0.23	1 [0.3–2]	1.1 [0.4–1.1]	0.90	0.3 [0.3–0.4]	0.4 [0.3–1.1]	0.02
**Time from molecular assay results to discharge** *									
**median [IQ25–IQ75], hours** b	24 [(−19)−43]	22 [19–42]	0.96	1.2 ([−4)−5]	−0.3 [(−5)−1]	0.31	21 [10–57]	19 [11–21]	0.26
**Administration of antibiotics**									
** No. of patients treated/total (%)**	5/8 (62.5)	7/17 (41.2)	0.41	8/45 (17.8)	2/23 (8.7)	0.48	9/16 (56.3)	14/33 (42.4)	0.54
** Length of treatment, median [IQ25–IQ75], days**	2 [2–2]	1 [1]–[2]	0.03	1.3 [1–2.5]	0.8 [0.5–1]	0.09	3 [1]–[4]	1 [1]–[2]	0.04
**Administration of acyclovir**									
** No. of patients treated/total (%)**	0/8	2/17 (11.8)	1.00	3/45 (6.7)	0/23	0.55	8/16 (50)	16/33 (48.5)	1.00
** Length of treatment, median [IQ25–IQ75], days**	/	1 [1–1]	NA	2 [1]–[5]	/	NA	2.5 [1]–[3]	1 [1]–[2]	0.40
**No. of patients who received computer** **tomographic scan/total (%)**	0/8	0/17		5/45 (11.1)	1/23 (4.3)	0.66	11/16 (68.8)	14/33 (42.4)	0.13
**Length of stay (LOS), median [IQ25–IQ75], days**	3.7 [2.6–4.1]	2.6 [2.2–3.1]	0.08	1.7 [1.2–2]	1 [0.9–1.5]	0.004	4 [2.4–5.2]	2 [1.8–3.5]	0.02

a Reference study [3].

b Only the patients (infants. children. and adults) admitted to hospital between Sunday 10 a.m. and Friday 10 a.m. were included (n = 98).

* i.e. time from event 1 to event 2 = event 2 - event 1, negative values meaning event 2 occurred before event 1.

e.g. time from results to discharge equal to −0.3 hr means that results were available 0.3 h after patients’ discharge.

On admission, 23 patients (31.5%) received at least one dose of intravenous antibiotics. Administration of antibiotics was significantly more frequent (p = 0.01) in infants (7/17, 41.2%) and adults (14/33, 42.4%) than in children (2/23, 8.7%). For two adults and one child, a single dose was administered in the emergency department. Once a positive EV molecular test was communicated, antibiotics were discontinued in 43% of adults (6/14) and infants (3/7). Two adults with meningitis symptoms and vertigo, and one adult with a sore throat continued to be treated for 3 to 4 days. Acyclovir was initiated in 18 patients, of whom 16 (88.9%) were adults, with a median duration of 1 day per patient [1]–[2]. For three (19%) adults, a single dose was administered in the emergency department, and acyclovir was discontinued in nine adults (56%) after HSV1/2 PCR and EV molecular tests results were available. Among the patients treated with acyclovir, seven adults had neurological signs such as vertigo and paresthesia associated with meningitis symptoms, and two patients were whiny infants with paresthesia. CT scan was carried out more frequently in adults (42.4%) than in children (4.3%) or infants (0%) (p = 0.0001), and was normal in all cases. All the patients recovered, and no serious neurological complications were observed.

### Comparison of the Management of Patients with EV Meningitis Over Two Periods

We compared the virological diagnosis and clinical management of patients with EV meningitis over the periods 2008–09 and 2005 (Table 1). The median TAT of the EV tests and the test-to-discharge time for patients were unchanged between the two periods after restricting the analysis to admissions made between Sunday and Friday morning (n = 98). In adults, the median TAT [0.4 days (IQ: 0.3–1.1)] in 2008–09 was slightly higher (p = 0.02) than that in 2005 [0.3 days (IQ: 0.3–0.4)]. If we restrict our population to patients for whom the molecular test results were available before discharge, the time to discharge was significantly shorter in adults in 2008–09 (20 hr [19–49]) than in 2005 (33 hr [21–69]) (p = 0.05), and tended to be shorter between the two periods in children (1.5 hr [1]–[3]) vs 3.8 hr [2–21], p = 0.09), and in infants (23 hr [20–42]) vs 43 hr [23–76], p = 0.12). There was a decrease in the prescription of antibiotics for all patients, but not to a level of significance (OR [95%CI] for adults 0.6 [0.2–1.9]; children: 0.4 [0.1–2.2]; and infants: 0.4 [0.1–2.3]). The median duration of antibiotic treatment per patient was reduced significantly in adults (1 day in 2008–09 vs 3 days in 2005; p = 0.04), and by one-half in infants (1 day in 2008–09 vs 2 days in 2005; p = 0.03). The prescription of acyclovir and the use of CT scan did not differ significantly between the two periods in all age groups. The median LOS was significantly decreased, by 2 days (p = 0.02) for adults, and 0.7 days (p = 0.004) for children between 2005 and 2008–09, and was also lower in infants (1.1 days, p = 0.08).

Clinically, there was no significant difference in the rates of headache, fever, vomiting between adults admitted in 2005 and those admitted in 2008–09 (Table S1). Similar results were obtained in children between the two periods. However, a significantly higher rate of photophobia in adults (37.5% in 2005 vs 82% in 2008–09, p = 0.0033), nuchal rigidity in children (58% in 2005 vs 87% in 2008–09, p = 0.026), and the association of fever, headache and nuchal rigidity in children (49% in 2005 vs 74% in 2008–09, p = 0.011) was observed.

Prolonged hospitalization >2 days was observed in 17/33 adults in 2008–09: 3 patients had a post-lumbar puncture headache, 5 had vertigo, 1 had swelling of the arm as the result of intravenous infusions, 1 had just returned from a trip to Madagascar, and 4 had received an antibiotic treatment before hospitalization. In 2005, 13/16 adults were hospitalized >2 days: 3 for vertigo, 3 with the presence of concomitant infection (urinary, otitis, and gastroenteritis), 3 with headache for more than 5 days. In the remaining 4 patients, nothing abnormal was reported in the clinical history.

### Factors Associated with the Length of Stay

The comparative analysis of patient management over the two observational study periods showed a significant decrease in LOS in adults and children. The relationships between LOS and different factors, including time parameters (TAT, discharge, EV season), biological data (CSF pleocytosis), and treatment use were further investigated by univariate and multivariate analyses gathering all age groups in both study periods.

Univariate analysis showed that the test-to-discharge time (p = 0.0001), the use of antiviral treatment (p = 0.04), and the use of CT scan (p = 0.001) were associated with longer LOS in children (Table 2). These factors, except antiviral treatment, were significantly associated with LOS in multivariate analysis. Acyclovir was prescribed to three children in our study (Table 2). One child aged 8 years was hospitalized for eight days for convulsion and left-sided hemiplegia. The other two children, aged 4 years, were admitted for vomiting and headache and respiratory tract manifestations, which had been present for more than 3 days. Analysis of CSF of these two patients showed a predominance of lymphocytes (>90%). The period outside the peak of EV prevalence as well as CSF pleocytosis were not associated with a longer LOS in children.

**Table 2:** Univariate and multivariate analysis of factors associated with hospital length of stay in children (n = 68) with enterovirus (EV) meningitis during the two study periods (2005–2008–09).

Variable	Median days	Univariate analysis			Multivariate analysis		
	(IQ25–IQ75)	Hazard Ratio^a^	95% CI	P-value	Hazard Ratio^a^	95% CI	P-value
**Time parameters**							
Turnaround time (TAT)							
Results on the day (n = 26)	1.2 (0.9–1.7)	Ref		0.36			
Results within 24 h (n = 20)	1.7 (1.3–2.0)	0.58	0.31–1.08	0.09			
Results within 48 h (n = 15 )	1.4 (1.0–2.2)	0.72	0.37–1.42	0.35			
Results >48 h (n = 7)	1.0 (0.9–2.0)	0.9	0.37–2.18	0.82			
Time from molecular EV assay results to discharge							
Discharge in 5 h after results (n = 21)	1.3 (1.1–1.9)	Ref		<0.0001			<0.0001
Discharge within 6–24 h after results (n = 9)	1.9 (1.7–2.1)	0.51	0.25–1.03	0.06	0.38	0.16–0.95	0.04
Discharge >24 h after results (n = 3)	6.6 (3.6–7.6)	0.05	0.01–0.24	<0.0001	0.05	0.01–0.24	<0.0001
Discharge before results (n = 35)	1.1 (0.9–1.9)	1.28	0.74–2.23	0.38	1.23	0.74–2.04	0.44
Patients presenting outside EV season (n = 14)	1.7 (1.0–2.0)	Ref					
Patients presenting during EVseason (n = 54)	1.3 (1.0–1.9)	1.21	0.73–2.02	0.46			
**CSF data**							
Samples without pleocytosis (n = 10)	1.6 (1.0–2.0)	Ref					
Samples with pleocytosis (n = 58)	1.4 (1.0–1.9)	1.34	0.67–2.7	0.41			
**Interventions**							
No antibiotic treatment required (n = 58)	1.3 (1.0–1.9)	Ref					
Antibiotic treatment required (n = 10)	1.5 (1.3–2.2)	0.78	0.43–1.42	0.42			
No aciclovir treatment required (n = 65)	1.3 (1.0–1.9)	Ref					
Aciclovir treatment required (n = 3)	3.1 (1.6–7.6)	0.24	0.06–0.93	0.04			
No computer tomographic scan (n = 62)	1.3 (1.0–1.9)	Ref					
Computer tomographic scan required (n = 6)	2.7 (2.0–3.0)	0.30	0.15–0.61	0.001	0.22	0.10–0.48	<0.0001

a Data show an adjusted hazard ratio (HR) with 95% CI through a simple and multiple Cox regression model. with HR over 1 denoting a decrease in LOS.

Abbreviations: CI. confidence interval; IQR. interquartile range. The enterovirus season was defined as May 1 through October 31 for each study period.

The test-to-discharge time was significantly associated with LOS in univariate and multivariate analyses (p = 0.002) in infants (Table 3). Gender (male) (p = 0.006), and hospitalization outside the peak EV prevalence (from November 1 to April 30) (p = 0.006) were also associated with a longer LOS (1 day).

**Table 3:** Univariate and multivariate analysis of factors associated with hospital length of stay in infants (n = 25) with enterovirus (EV) meningitis during the two study periods (2005–2008–09).

Variable	Median days	Univariate analysis			Multivariate analysis		
	(IQ25–IQ75)	Hazard Ratio^a^	95% CI	P-value	Hazard Ratio^a^	95% CI	P-value
**Time parameters**							
Turnaround time (TAT)							
Results on the day (n = 7)	2.8 (1.9–3.9)	Ref		0.26			
Results within 24 h (n = 9)	2.7 (2.3–3.5)	1.24	0.45 –3.44	0.68			
Results within 48 h (n = 6)	2.6 (2.0–3.1)	1.96	0.66–5.87	0.23			
Results >48 h (n = 3)	4.3 (1.7–8.2)	0.34	0.06–1.97	0.23			
Time from molecular EV assay results to discharge							
Discharge in 5 h after results (n = 3)	2.2 (1.8–2.3)	Ref		0.0001			0.002
Discharge within 6–24 h after results (n = 9)	2.7 (2.3–3.1)	0.27	0.11–0.69	0.006	0.51	0.10–2.58	0.42
Discharge >24 h after results (n = 8)	3.7 (3.0–4.3)	0.08	0.03–0.25	<0.0001	0.05	0.01–0.31	0.002
Discharge before results (n = 5)	2 (1.7–3.1)	0.24	0.04–1.43	0.12	0.69	0.12–3.97	0.67
Patients presenting outside EV season (n = 3)	3.7 (3.1–4.3)	Ref					
Patients presenting during EV season (n = 22)	2.7 (2.2–3.4)	1.85	1.00–3.45	0.05	7.72	1.80–33.16	0.006
**CSF data**							
Samples without pleocytosis (n = 5)	2.3 (2.3–2.8)	Ref					
Samples with pleocytosis(n = 20)	3 (2.2–3.6)	1.14	0.27–4.71	0.86			
**Interventions**							
No antibiotic treatment required (n = 13)	2.5 (2.2–3.1)	Ref					
Antibiotic treatment required (n = 12)	3.1 (2.5–3.8)	0.66	0.29–1.47	0.31			
No aciclovir treatment required (n = 23)	2.9 (2.2–3.7)	Ref					
Aciclovir treatment required (n = 2)	2.8 (2.7–2.8)	1.58	0.85–2.91	0.15			

a Data show an adjusted hazard ratio (HR) with 95% CI through a simple and multiple Cox regression model. with HR over 1 denoting a decrease in LOS.

Abbreviations: CI. confidence interval; IQR. interquartile range.The enterovirus season was defined as May 1 through October 31 for each study period.

Multivariate analysis showed that TAT of the EV test was correlated with LOS of adults, because every 24-hour increase in TAT was associated with a mean 45% increase in LOS (Table 4). Of the 49 adults, 39 (80%) were discharged only after positive results of the EV molecular test were available. In the univariate and multivariate analyses the test-to-discharge time had a significant effect on LOS (p = 0.0001). A longer median LOS was observed in adults treated with antibiotics (3.3 vs 1.9 days, LogRk *p* = 0.17 vs Wilcoxon *p* = 0.008) and acyclovir (3.2 vs 2 days, LogRk *p* = 0.28 vs Wilcoxon *p* = 0.06) than in untreated patients. The median duration of acyclovir treatment (3 days [0–5]) was correlated with LOS (SCC = 0.5, *p* = 0.01, data not shown).

**Table 4:** Univariate and multivariate analysis of factors associated with hospital length of stay in adults (n = 49) with enterovirus (EV) meningitis during the two study periods (2005–2008–09).

Variable	Median days	Univariate analysis			Multivariate analysis		
	(IQ25–IQ75)	Hazard Ratio^a^	95% CI	P-value	Hazard Ratio^a^	95% CI	P-value
**Time parameters**							
Turnaround time (TAT)							
Results on the day (n = 26)	2 (1.7–3.1)	Ref		0.19			<0.0001
Results within 24 h (n = 11)	3.1 (2.0–6.2)	0.49	0.21–1.19	0.11	0.15	0.05–0.44	<0.0001
Results within 48 h (n = 8 )	3.8 (2.8–4.7)	0.57	0.31–1.06	0.07	0.17	0.07–0.42	<0.0001
Results >48 h (n = 4)	4.4 (2.9–4.9)	0.52	0.27–1.01	0.05	0.01	0.002–0.07	<0.0001
**Time from molecular EV assay results to discharge**							
Discharge in 5 h after results (n = 2)	1.7 (1.2–2.2)	Ref		0.0001			<0.0001
Discharge within 6–24 h after results (n = 21)	2 (1.8–3.3)	0.45	0.14–1.38	0.16	0.14	0.07–0.28	<0.0001
Discharge >24 h after results (n = 16)	4.6 (3.6–6.1)	0.10	0.03–0.32	<0.0001	0.01	0.003–0.03	<0.0001
Discharge before results (n = 10)	1.3 (0.7–2.0)	0.76	0.17–3.33	0.71	1.58	0.62–4.00	0.34
Patients presenting outside EV season (n = 7)	3.6 (2.6–5.0)	Ref					
Patients presenting during EV season (n = 42)	2.3 (1.8–4.0)	1.39	0.75–2.55	0.29			
**CSF data**							
Samples without pleocytosis (n = 12)	2.4 (1.8–4.6)	Ref					
Samples with pleocytosis (n = 37)	3.0 (1.8–4.0)	1.17	0.59–2.34	0.66			
**Interventions**							
No antibiotic treatment required (n = 26)	1.9 (1.3–3.9)	Ref					
Antibiotic treatment required (n = 23)	3.3 (2.2–4.8)	0.63	0.36–1.11	0.11			
No aciclovir treatment required (n = 25)	2 (1.7–4.0)	Ref					
Aciclovir treatment Required (n = 24)	3.2 (2.1–4.0)	0.86	0.49–1.51	0.59			
No computer tomographic scan (n = 24)	2.3 (1.8–3.6)	Ref					
Computer tomographic scan required (n = 25)	3.1 (1.7–5.0)	0.62	0.34–1.13	0.12			

a Data show an adjusted hazard ratio (HR) with 95% CI through a simple and multiple Cox regression model. with HR over 1 denoting a decrease in LOS.

Abbreviations: CI. confidence interval; IQR. interquartile range. The enterovirus season was defined as May 1 through October 31 for each study period.

For all age groups, the median duration of antibiotic treatment was significantly correlated with LOS: children (1 day [0.5–4.5], SCC = 0.9, *p* = 0.001), infants (2 days [1]–[6], SCC = 0.6, *p* = 0.03), and adults (1 day [range, 0.5–7], SCC = 0.6, *p* = 0.003). Antibiotic administration was immediately stopped after a positive result of EV molecular test in 4/10 children (40%), 5/12 infants (42%), and 9/23 adults (39%). Finally, regardless of patient age, CSF pleocytosis was not associated with a longer LOS.

## Discussion

This study reports the assessment of professional practices in the management of EV meningitis in three age groups of patients admitted to hospital over observational study periods in 2005 and 2008–09. Various factors were in favor of such a comparison. The study involved the same clinical departments and over both periods physicians’ real-life daily practice was analyzed without any prior warning or experimental protocol. In addition, the conditions of virological molecular diagnosis were the same during the two study periods, involving a single laboratory and the same team (technicians and biologists). Thus, it can be assumed that any bias was minimized. We observed a significantly shorter hospital LOS in both adults and children, and a shorter duration of antibiotic use in adults and infants, which indicates an improvement in patient management between the two periods. As the technical time of the molecular assays was similar during the two study periods, we made progress in reporting virological results to physicians, owing to a better organization of the analytical phase and the work schedule in the laboratory. The results of the EV molecular testing procedure were obtained within 24 hours for 92% of the 2008–09 patients and for only 70% of the 2005 patients (p = 0.002). Although efforts are made continuously to achieve a satisfactory degree of analytical quality, there is evidence that the preanalytic conditions, including sample preparation and sample transport from the clinical ward to the laboratory, are crucial and can influence patient management. As adult hospitalization units are located in the vicinity of our virology laboratory, CSF specimens are rapidly available for EV testing and consequently the results are communicated the same day to the physician (70% of patients). In contrast, only 40% of children benefited from the results of our EV molecular test on the same day because pediatric units are a 30 minutes’ drive away from the laboratory.

Earlier studies showed that the introduction of a rapid EV molecular test and the availability of positive EV results were associated with shorter LOS and/or duration of antibiotic use [1], [3], [4], [9], [10], [14]. Our study shows that a positive EV molecular test result has a different clinical impact according to the age group of patients. Numerous factors can explain these differences, including knowledge about EV meningitis, physician experience, and precautionary measures.

The greatest value of the prospective diagnosis of EV meningitis was observed in adults, a population not often studied in other reports. Their inclusion in our study is a major novel feature. The improvement in the clinical management of the adults between the two study periods could have been due to a combination of different factors. First, multivariate analysis showed that TAT was correlated with LOS, hence every 24-hour increase in TAT was associated with a mean 45% increase in LOS. Second, greater physician responsiveness in the postanalytic phase, after communication of positive EV results, was also observed, and resulted in the discharge of adult patients 20 hours later compared to 33 hours in 2005 (p = 0.05). The adults were hospitalized on four different clinical wards, and as yet patient management is not fully standardized. Third, the therapeutic management of adults also evolved since antibiotic treatment was discontinued significantly earlier, with a reduction in median treatment duration of 2 days in 2008–09 compared to that in 2005. Antibiotics were immediately stopped in 43% of cases after a positive EV result, and 30% of all patients received just one dose during the period 2008–09. Interestingly, the rate of antibiotic prescription in adult medical wards of our hospital remained lower than that reported in the literature, which varies from 66 to 80% [15], [16]. However, no significant difference was observed in the rates of meningitis symptoms in adults between the two periods.

In adults, the frequency of EV meningitis is still probably somewhat underestimated and under-investigated [2], which may delay ordering of the EV molecular test. In our hospital, the number of EV diagnostic tests in adults increased significantly between 1999 and 2009. During this 10-year period 28% of diagnosed cases of EV meningitis were observed in adults [2, 3, and 5]. These studies show that professional practice improved over time since EVs are now more often looked for and the results taken into consideration.

In children, EV meningitis has long been recognized by pediatricians as a common, usually benign disease, characterized by rapid recovery. Empiric antibiotic treatment was given to only 9% of children in our hospital in 2009, compared to 76–100% in other studies [4], [17], good evidence of the extensive experience of the pediatricians taking part in our study. Being certain of obtaining results by the end of the day, these pediatricians discharged more than one child in two from the hospital before receiving the results of the positive EV molecular test. As a result these children were managed on an outpatient basis.

Our laboratory currently provides molecular testing 5 days a week. Diagnosis could perhaps be improved by performing tests on all 6 working days. However, in the study of Cohen-Bacrie et al. [14], the establishment of a mini lab (point of care (POC)) in the vicinity of the pediatric emergency ward, allowing 24-hour molecular EV testing every day, did not decrease the children’s hospital LOS.

In infants, multivariate analysis showed that TAT of EV testing was not associated with LOS, which is consistent with findings reported in two earlier studies [1], [14]. Patients were not immediately discharged following a positive result of the EV test but within a median time of 23 hr. Later discharge is considered as a precautionary measure because pediatricians prefer to wait for the results of bacteriological tests (urinary analysis and CSF culture), which are obtained within 24 to 48 hr, since fever is often the only symptom. Nevertheless, empiric antibiotic treatment was discontinued in 43% of infants after positive EV results, which is attributable to the molecular assay and the importance attached to it by the clinicians in charge of the infants. If infants were admitted other than during the peak of EV prevalence their management was significantly altered, with multivariate analysis showing that LOS increased by one day. This result needs to be confirmed with a larger number of infants. Our records over a period of more than 10 years show that 30% of all cases of EV meningitis were diagnosed outside the EV season (data not shown).

This study has certain limitations. First, it includes results from a single institution over two study periods. However, it is possible that the same results could be obtained in hospitals in towns of a similar size, with a similar technical and clinical set up to ours, and with a virology laboratory on site. Second, the age distribution changed between 2005 and 2008–09, with more adults in 2008–09.

In conclusion, this study afforded an opportunity to evaluate professional practices within our institution and to identify factors associated with the clinical management of patients with EV meningitis. The clinical impact of the positive EV molecular results varied according to the age group of patients. In infants, a positive result led to antibiotics being discontinued in 43% of cases. In children, greater physician responsiveness resulted in shorter LOS. Because LOS of children is shorter we can do no more to improve the technical EV diagnosis. In adults, we found a direct correlation between test turnaround time and LOS and a significant reduction in the time to discharge after positive results in 2008–09. The French health policy aimed at reducing the duration of hospitalization and the prescription of antibiotics may also have contributed to the improvement of the management of meningitis in the 2008–09 period. Finally, experience in handling EV meningitis, and close communication between physicians and the virology laboratory in daily practice remain essential to the improvement of clinical management of patient.

## Supporting Information

Table S1 Clinical characteristics of patients on admission over the two study periods (2005 vs 2008–09).
(PDF)
