# Supplementary material for: Improvement of the Management of Infants, Children and Adults with a Molecular Diagnosis of Enterovirus Meningitis during Two Observational Study Periods
Source: PLoS One. 2013 Jul 11;8(7):e68571. doi: 10.1371/journal.pone.0068571 (PMC3708915; doi:10.1371/journal.pone.0068571)
Supplement: Table S1 — Clinical characteristics of patients on admission over the two study periods (2005 vs 2008–09). (PDF) [file pone.0068571.s001.pdf]

Table S1. Clinical characteristics of patients on admission over the two study periods (2005 vs 2008-09)[a]

**Clinical characteristics**	**Adults**			**Children**			**Infants**		
	**2005** n=16	**2008-09** n=33	**P-value**	**2005** n=45	**2008-09** n=23	**P-value**	**2005** n=8	**2008-09** n=17	**P-value**
Fever	13 (81)	26 (79)	1.00	36 (44)	20 (87)	0.73	8 (100)	16 (94)	1.00
Headache	15 (94)	33 (100)	0.32	40 (89)	23 (100)	0.15			
Nuchal rigidity	5 (31)	19 (58)	0.13	26 (58)	20 (87)	0.026			
Photophobia	6 (38)	27 (82)	0.003	19 (42)	15 (65)	0.12			
Vomiting	10 (63)	27 (67)	1.00	34 (76)	21 (91)	0.19	0	5 (29)	0.14
Association fever, headache and nuchal rigidity	4 (25)	16 (49)	0.14	18 (40)	17 (74)	0.011			

[a] : Values are the number (percentage) of patients with the indicated characteristics
